# Metagenomic nanopore sequencing for exploring the nature of antimicrobial metabolites of *Bacillus haynesii*

**DOI:** 10.1186/s13568-024-01701-8

**Published:** 2024-05-04

**Authors:** Mohamed A. Eltokhy, Bishoy T. Saad, Wafaa N. Eltayeb, Mohammad Y. Alshahrani, Sahar M R Radwan, Khaled M. Aboshanab, Mohamed S. E. Ashour

**Affiliations:** 1 Department of Microbiology, Faculty of Pharmacy, Misr International University (MIU), Cairo, 19648 Egypt; 2 Department of Bioinformatics, HITS Solutions Co., Cairo, 11765 Egypt; 3 Department of Clinical Laboratory Sciences, College of Applied Medical Sciences, King Khalid University, P.O. Box 61413, Abha, 9088 Saudi Arabia; 4 Department of Microbiology and Immunology, Faculty of Pharmacy, Al-Azhar University (Girls), Organization of African Unity St., Cairo, 11651 Egypt; 5 Department of Microbiology and Immunology, Faculty of Pharmacy, Organization of African Unity St, Ain Shams University, Organization of African Unity St., Cairo, 11566 Egypt; 6 Department of Microbiology and Immunology, Faculty of Pharmacy, Al-Azhar University (Boys), Cairo, 11651 Egypt

**Keywords:** *Bacillus haynesii*, Metagenomic, Soil, Nanopore sequencing, Multidrug-resistant

## Abstract

Multidrug-resistant (MDR) pathogens are a rising global health worry that imposes an urgent need for the discovery of novel antibiotics particularly those of natural origin. In this context, we aimed to use the metagenomic nanopore sequence analysis of soil microbiota coupled with the conventional phenotypic screening and genomic analysis for identifying the antimicrobial metabolites produced by promising soil isolate(s). In this study, whole metagenome analysis of the soil sample(s) was performed using MinION™ (Oxford Nanopore Technologies). Aligning and analysis of sequences for probable secondary metabolite gene clusters were extracted and analyzed using the antiSMASH version 2 and DeepBGC. Results of the metagenomic analysis showed the most abundant taxa were *Bifidobacterium, Burkholderia*, and *Nocardiaceae* (99.21%, followed by *Sphingomonadaceae* (82.03%) and *B. haynesii* (34%). Phenotypic screening of the respective soil samples has resulted in a promising *Bacillus* isolate that exhibited broad-spectrum antibacterial activities against various MDR pathogens. It was identified using microscopical, cultural, and molecular methods as *Bacillus (B.) haynesii* isolate MZ922052. The secondary metabolite gene analysis revealed the conservation of seven biosynthetic gene clusters of antibacterial metabolites namely, siderophore lichenicidin VK21-A1/A2 (95% identity), lichenysin (100%), fengycin (53%), terpenes (100%), bacteriocin (100%), Lasso peptide (95%) and bacillibactin (53%). In conclusion, metagenomic nanopore sequence analysis of soil samples coupled with conventional screening helped identify *B. haynesii* isolate MZ922052 harboring seven biosynthetic gene clusters of promising antimicrobial metabolites. This is the first report for identifying the bacteriocin, lichenysin, and fengycin biosynthetic gene clusters in *B. haynesii* MZ922052.

**Supplementary Information:**

The online version contains supplementary material available at 10.1186/s13568-024-01701-8.

**Key points**

1. Metagenomic analysis of soil samples coupled with conventional screening is of help to explore the nature of various antimicrobial metabolites.
2. This study resulted in the isolation of a *Bacillus haynesii* isolate MZ922052 that exhibited broad-spectrum antibacterial activities against various MDR pathogens.
3. Secondary metabolite gene analysis revealed the conservation of biosynthetic gene clusters of siderophore lichenicidin VK21-A1/A2, lichenysin, fengycin, terpenes, bacteriocin, Lasso peptide and bacillibactin.
4. This is the first report for identifying the biosynthetic gene clusters of bacteriocin, lichenysin, and fengycin biosynthetic gene clusters in *B. haynesii* isolate, MZ922052.

## Introduction

 Antimicrobial resistance (AMR) is now a worldwide health crisis where antibiotics are increasingly reaching a point where they can no longer effectively treat infections (Hu et al. 2020). Nowadays, there is a decrease in pharmaceutical industry investment in discovering novel antibiotics, triggering, the threat of antibiotic resistance (Chinemerem Nwobodo et al. 2022). There will always be an urgent need for novel antibiotics. Soil harbors many microorganisms that are good sources of antimicrobials and antibiotics. These are shown to be a hopeful source for novel antimicrobials (Polianciuc et al. 2020). Many new antimicrobials have been extracted and fully distinguished from soil bacteria and other diverse natural habitats (Hallaj-Nezhadi et al. 2022). Natural soils are known for their biodiversity and are the leading supplier of possible novel antibiotics (Amin et al. 2015).

*Bacillus* is a genus characterized by its heterogeneity of bacteria with species producing tremendous antimicrobial metabolites that cure various microbial infections. *Bacillus* sp. are the most considerable bacterial strains found on earth. They are Gram-positive and endospore-forming bacteria (Rampersad and Ammons 2005; Hallaj-Nezhadi et al. 2022; Vehapi et al. 2023; Caulier et al. 2019). Many studies have been conducted to extract antimicrobial compounds from different strains of *Bacillus* and characterize these antimicrobials (Berić et al. 2014;Vehapi et al. 2023;.Caulier et al. 2019).

*Bacillus* genera have many heterogeneous species that produce antimicrobial compounds. Most members of this genus are antibiotic producers. These antibiotics are mostly low-molecular-weight peptides that exhibit antitumor, antibacterial, and antiviral activities Caulier et al. 2019). The antibiotic bacitracin has been known to be synthesized by *B. licheniformis* and *B. subtilis* which is known for its efficacy against Gram-positive bacteria (Johnson et al. 1945; Haavik and Froyshov 1975). One of the most important species is *B. licheniforms* (Saggese et al. 2022). Antimicrobial metabolites that are produced and extracted from *Bacillus* sp. which are inhabitants of the natural environment, such as soil, provide a substantial role in preventing and curing microbial diseases and are shown to be a leading source of novel antimicrobials (Polianciuc et al. 2020).

Bioinformatics has been a valuable tool for mining genes that produce antimicrobials from soil bacteria (Baltz 2021). Bacterial genome mining is a bioinformatic way to discover the biosynthesis of antimicrobial genes in the genome of bacteria. Computational algorithms approach genome mining to analyze secondary metabolite gene clusters (Baltz 2021). Therefore, it is important to apply metagenomic analysis to secondary metabolite gene clusters for the discovery of novel antimicrobials. Previous studies conducted in our lab where metagenomic nanopore sequencing has been undertaken to determine the biosynthetic gene clusters involved in the biosynthesis of certain functioning metabolites of *Alcaligenes faecalis* and *Paenibacillus ehimensis* soil isolates (Eltokhy et al. 2021a, b). Such analysis was coupled with conventional screening and advanced spectroscopic analysis to identify the nature and chemistry of the respective metabolites (Eltokhy et al. 2021a, b). It was found that combining these techniques was found to be helpful and accurate in rapidly identifying the various active metabolites produced by the respective soil isolates (Eltokhy et al. 2021a, b). Therefore, this study aimed to use metagenomic analysis of the soil samples in combination with the conventional phenotypic screening to identify the promising antimicrobial-producing soil isolate(s) and to explore the nature of potential secondary metabolites produced by the respective soil isolate(s).

## Materials and methods

### Whole metagenome analysis of the soil sample

#### DNA extraction and quantification

DNA extraction was done by Qiagen DNeasy power-soil kit (Qiagen, Hilden, Germany) according to the producer’s recommendations. DNA concentration was determined by Qubit fluorometer ver. 4.0 (Thermo Fisher Scientific, Waltham, Massachusetts, USA) to ensure there is not less than 55 ng/µL of DNA, as stated in Oxford nanopore Standard operating procedure (Eltokhy et al. 2021a).

### Library construction

To 12 µL DNA, 34 µL sequencing buffer, 25.5 µL of loading beads, and 4.5 µL nuclease-free water were included and mixed. Construction of the library was done by a Rapid Sequencing Kit (Oxford Nanopore Technologies, Oxford, UK). Priming and loading onto the FLO-MIN106 (Nanopore Technology, Oxford, England) flow cell were performed after library construction (Eltokhy et al. 2021a).

### Sequencing and data analysis

MinION™ (Oxford Nanopore Technologies, Oxford, UK) was applied for running sequences. Twelve hours generate 3.03 M reads with N50 equals 9.29 K. Real-time base calling during sequencing was generated by the Guppy software. The output was in the form of FAST5 and FASTq files, reads below Q7 were excluded. Classification of sequences to taxonomic identifiers was generated by Centrifuge software (Kim et al. 2016)(Kim et al. 2016). Bacterial and viral genomes, as well as human reference genome (GRCh38) downloaded from the National Center of Biotechnology Information (NCBI) RefSeq were used for the construction of the Centrifuge index. Dust masker (v1.0.0, NCBI) was applied for masking low-complexity regions with a dust score greater than 20 in the reference sequences. Re-centrifuged was applied for visualization of results (Martí 2019).

### Extraction of secondary metabolites from genome sequences

Aligning and analysis of sequences for probable secondary metabolite gene clusters were extracted by antiSMASH version 2 (Antibiotics and Secondary Metabolite Analysis Shell) (https://antismash.secondarymetabolites.org/#!/start (accessed on 10 December 2023). The genomic sequence was further assembled and analyzed using deepBGC (https://github.com/Merck/deepbgc (accessed on 17 March 2024) (Hannigan et al. 2019). Draft genome comparison was done by applying Mauve software (https://gel.ahabs.wisc.edu/mauve) (accessed on 12 December 2023) (Kapley et al. 2016).

### Cheminformatic analysis of the detected secondary metabolites

Cheminformatic analysis of the detected secondary metabolites including the 2D structure and molecular weight analysis was evaluated using PubChem 2.1 database (https://pubchem.ncbi.nlm.nih.gov/ (accessed on 10 January 2024) as previously reported (Kim et al. 2023).

### Bacterial isolation and antimicrobial screening

A *Bacillus* isolate coded SS10 was isolated from the tested soil as previously described (Eltokhy et al. 2021a). Briefly, the collected soil sample was placed in a hot air oven and heated at 80^°^C for one hour Ince E 2008). About 9 mL saline tube was inoculated with 1 gm soil, and then vortexed for 4 min at 400 rpm Rampersad and Ammons 2005) followed by a 10-fold serial dilution was done within the range of 10^− 1^ to 10^− 6^. One mL of each dilution was transferred to the surface of Starch Casein Agar (SCA) and incubated for 7 days (Ranjan and Jadeja 2017). Different colonies were picked from the SCA, and a preliminary screening was performed as previously reported (Eltokhy et al. 2021a, b).

A pure bacterial isolate was screened for antimicrobial activity by agar well diffusion method against three standard strains of *E. coli* ATCC 25,922, *S. aureus* ATCC 25,293, and *C. albicans* ATCC 10,231. The test was also performed against clinical isolates including, three vancomycin-resistance *S. aureus* (VRSA1, VRSA2, and VRSA3), *Staphylococcus (S.) epidermidis* (SE1, SE2, SE3), three MDR *K. pneumoniae* (KP1, KP2, and KP3), two MDR *E. coli* (EC1 and EC2), *Candida albicans*, (CA1, CA2) and *Candida auris* (CS1, CS2). These clinical isolates were provided by the Central Microbiology Lab of Ain Shams Hospital, Cairo, Egypt of anonymous discharged patient samples. The clinical isolates were isolated in the hospital lab for routine checkups of culture and sensitivity. The Faculty of Pharmacy Ain Shams University Ethics Committee Number, ACUC-FP-ASU -REC# 75 approved the study.

The antibiogram of the clinical bacterial isolates was evaluated using the Kirby-Bauer method, against various antibiotic discs (ThermoScientific™ and Oxoid™, MA, USA) according to Clinical Laboratory Standard Institute (CLSI) guidelines 2021 (CLSI 2021). The vancomycin susceptibility test was evaluated using the agar dilution method (resistant isolates, MIC ≥ 16 µg/mL for *S. aureus* and ≥ 32 µg/mL for other staphylococci) according to CLSI guidelines. Their antibiogram showed that the SE, SE2, and SE3 isolates of SE were resistant to clindamycin, gentamicin, cefoxitin, and ciprofloxacin. The three VRSA isolates were resistant to vancomycin and cefoxitin. The VRSA2 and VRSA3 were resistant to clindamycin, gentamicin, and ciprofloxacin. KP1, KP2, and KP3 were resistant to most of the tested antibiotics according to CLSI guidelines. EC1 was resistant to cefotaxime and imipenem only, while EC2 was resistant to most tested antibiotics. The standard strains, *E. coli* ATCC® 25,922 and *S. aureus* ATCC® 25,923 were employed for quality control (CLSI 2021).

### Identification of the isolated soil bacteria

The soil bacterial isolate was identified by biochemical reactions and DNA sequencing of 16 S ribosomal RNA. Sequencing and analysis of data for the isolate was done by Sigma Scientific Services Co., Egypt through GATC Biotech Co., Germany. The assembled contig of the 16 S ribosomal RNA was blasted and aligned by BLAST, https://blast.ncbi.nlm.nih.gov/Blast.cgi (accessed on 18 December 2023). The percentage homology between the sequence database and query sequence was provided and determined. The phylogenetic tree was constructed using Log-Expectation through Multiple Sequence Comparisons (MUSCLE, https://www.ebi.ac.uk/Tools/msa/muscle) (accessed on 18 December 2023) (Edgar 2004). Bootstrap analysis (1000 replicates) was applied for inferring phylogenetic trees. The 16 S ribosomal RNA sequence of the selected isolate was deposited in the NCBI GenBank (https://www.ncbi.nlm.nih.gov/).

### Deposition of *Bacillus* isolate SS10 a local culture collection

The molecularly identified *Bacillus* isolate SS10 was deposited in the Culture Collection Ain Shams University (CCASU), Cairo, Egypt as *Bacillus* isolate CCASU-SS10-32 (http://ccinfo.wdcm.org/collection/by_id/1186 (accessed on 30 December 2023).

### Shake flasks method for production of antimicrobial metabolite(s)

#### Preparation of the seed culture

Preparation of the seed culture was done as previously mentioned by(Eltokhy et al. 2021a), by transferring three loopfuls of 24 h bacterial culture into starch casein broth (50 mL) and incubated for 24 h at 35^°^C in a shaking incubator adjusted at 200 rpm. Centrifugation of 1 mL of the culture for 5 min at 16,000 rpm was applied using a microcentrifuge. The sedimented cells were washed twice with 1 mL sterile saline and inoculated in 20 flasks each containing 100 mL of casein starch broth. Incubation of the 20 flasks in a shaking incubator, adjusted at 150 rpm, for 7–10 days at 35^°^C (Ranjan and Jadeja 2017)

#### Extraction process

Sequential extraction of cell-free culture medium is carried out by using ethyl acetate and dichloromethane (1:1). Equal volumes of ethyl acetate and cell-free culture medium were added in a separating funnel and shaken thoroughly for 2 h for 10 min of intervals. The separating funnel was left overnight, and the ethyl acetate upper layer was separated and stored at 4^°^C. The same procedure was repeated for dichloromethane using the culture medium left after extraction with ethyl acetate Ranjan and Jadeja 2017). Ethyl acetate and dichloromethane extracts were dried at 45^°^C by a rotary evaporator (Buchi R205, Flawil, Switzerland) (Selvin et al. 2009; Ali et al. 2019; Rajaram et al. 2020). The residues left after evaporation were dissolved in 1 mL dimethylsulfoxide (DMSO) (Valan Arasu et al. 2009)and the agar well diffusion method was applied for the examination of the antimicrobial activities of the extracts.

#### Evaluation of the antimicrobial activities

The residues left after evaporation of either ethyl acetate or dichloromethane extracts were dissolved in 1 mL DMSO (100%). In addition, 0.5 mL of DMSO-residue extract (100%) was taken and diluted with 0.5 mL DMSO to obtain 50% DMSO-residue extract, and both were tested for antimicrobial activity by well agar diffusion where pure DMSO, ethyl acetate and dichloromethane were used as negative controls (Rajaram et al. 2020).

## Results

### Metagenomics of mud soil from a garden at Luxor, Egypt

The DNA sample was quantified by Qubit fluorometer to ensure it passes the cutoff value of 150% concentration of DNA material and OD 260 nm/280 nm ratio between 1.8 and 2.0 in the sample, as mentioned by the Oxford nanopore manual. A range between 500 and 1080 reads in the sample was obtained. The length of sequences ranged between 250 and 12,000 bp. No duplicate reads were observed or N count (ambiguous). Good quality FastQ files showed a score range between 10 and < 25 per base Phred. The percentage abundance of the taxa showed that the most abundant taxa were *Bifidobacterium, Burkholderia* and *Nocardiaceae* (99.21%). *Sphingomonadaceae* was second showing 82.03%. *B. haynesii* showed about 34% (Fig. S1). The metagenomics sequences were deposited in the NCBI GenBank sequence Archives under accession number PRJNA1064698 (https://www.ncbi.nlm.nih.gov/sra/PRJNA1064698 (accessed on 16 December 2024).

### Antimicrobial preliminary screening

Preliminary screening of the *Bacillus* isolate SS10 showed positive inhibition of the growth of all the tested bacterial clinical isolates (SE1, SE2, SE2, VRSA1. VRSA2, VRSA3, KP1, KP2, KP3, EC1, EC2 and only one *C. albicans* isolate CA1 (Table 1). The agar diffusion for evaluating the antimicrobial activities of ethyl acetate extract of *B. haynesii* isolate MZ922052 against MDR *K. pneumoniae* clinical isolate (KP1), *C. albicans* clinical isolate (CA1) and the dichloromethane extract against vancomycin resistant *Staphylococcus aureus* (VRSA1) and MDR *Escherichia coli* (EC1) using DMSO, ethyl acetate and dichloromethane as negative controls is depicted in Fig. S2.

**Table 1:** The recorded inhibition zones of dichloromethane or ethyl acetate extracts of *B. haynesii* isolate MZ922052 against various clinical and standard bacterial isolates

Mean zone of inhibition (mm) ± SD		
Tested Microorganisms	Dichloromethane extract	Ethyl acetate extract
Clinical isolates		
SE1, SE2, SE3	11 ± 0.5	16 ± 1.0
VRSA1, VRSA2, VRSA3	12 ± 0.5	14 ± 1.0
MDR EC1	11 ± 0.5	11 ± 0.5
MDR EC2	12 ± 1.0	12 ± 1.0
MDR KP1	12 ± 1.0	13 ± 0.5
MDR KP2	11 ± 1.0	14 ± 1.0
MDR KP3	11 ± 1.0	14 ± 1.0
CA1	12 ± 0.5	11 ± 0.5
Standard strains		
*C. albicans* ATCC 10,231	12 ± 0.5	16 ± 1.0
*S. aureus* ATCC 25,293	11 ± 1.0	13 ± 0.5
*E. coli* ATCC 25,922	12 ± 1.0	14 ± 0.5

MDR, multidrug-resistant; VRSA, vancomycin resistant Staphylococcus aureus; EC, Escherichia coli, KP, Klebsiella pneumoniae, CA, Candida albicans.SE, Streptococcus epidermidis; SD, standard deviation

### Identification of *Bacillus* isolate SS10

Based on microscopical, cultural and 16 S ribosomal DNA sequence alignment, the *Bacillus* isolate SS10 was identified as *B. haynesii* isolate MZ922052. The phylogenetic analysis of the *B. haynesii* isolate MZ922052 (Query) is displayed in Fig. S2. The 16 S ribosomal RNA gene sequence was deposited in the NCBI GenBank database under nucleotide accession number MZ922052.

### Antimicrobial evaluation of the extracted metabolite(s)

The ethyl acetate was the optimum solvent for extraction as the mean zone of inhibition of the tested bacteria ranged from 11 to 16 mm **±** 1.0 mm, while dichloromethane extract showed weaker inhibition zones (around 11 mm) as presented in Table 1. The antimicrobial activities of either dichloromethane or ethyl acetate extracts of *B. haynesii* isolate MZ922052 in terms of inhibition zones are displayed in Table 1.

### Characterization of the antimicrobial metabolite

#### Identification of the biosynthetic gene clusters using the antiSMASH

**Fig. 1:**
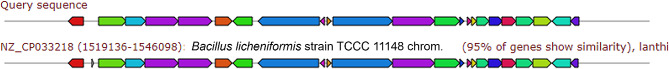
Biosynthetic gene cluster arrangement of the siderophore lichenicidin VK21-A1/A2 of Bacillus haynesii isolate MZ922052 (query sequence) compared to the homologous (95% identity) biosynthetic gene cluster of B. licheniformis using antiSMASH. Putative biosynthetic genes are presented in blue, additional biosynthetic genes in purple, transport, regulation-related genes in green, and resistance genes in red

**Fig. 2:**
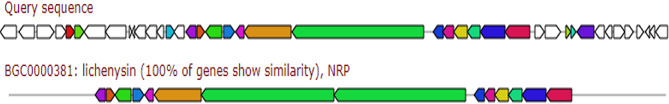
Biosynthetic gene cluster arrangement of lichenysin of *Bacillus haynesii* isolate MZ922052 (query sequence) compared to the homologous (100% identity) biosynthetic gene cluster of *B. licheniformis* using antiSMASH. Putative biosynthetic genes are presented in green, additional biosynthetic genes in brown, transport, and regulation-related genes in blue, and resistance genes in red

**Fig. 3:**
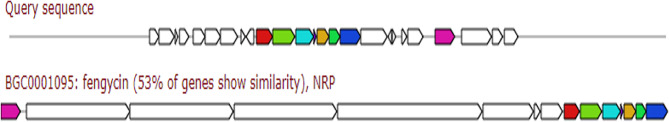
Biosynthetic gene cluster arrangement of fengycin of *Bacillus haynesii* isolate MZ922052 (query sequence) compared to the homologous (53% identity) biosynthetic gene cluster of *B. licheniformis* using antiSMASH. Putative biosynthetic genes are presented in green, additional biosynthetic genes in brown, transport, and regulation-related genes in blue, and resistance genes in red

**Fig. 4:**
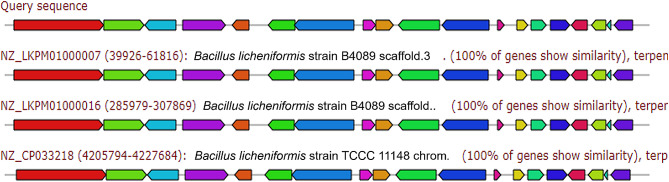
Biosynthetic gene cluster arrangement of terpenes of *Bacillus haynesii* isolate MZ922052 (query sequence) compared to the homologous (100% identity) biosynthetic gene cluster of three *B. licheniformis* strains using antiSMASH. Putative biosynthetic genes are presented in green, additional biosynthetic genes in brown, transport, and regulation-related genes in blue, and resistance genes in red

**Fig. 5:**
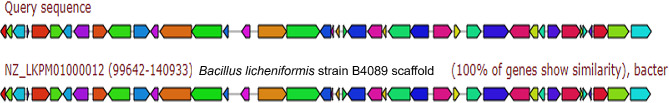
Biosynthetic gene cluster arrangement of bacteriocin of *Bacillus haynesii* isolate MZ922052 (query sequence) compared to the homologous (100% identity) biosynthetic gene cluster of *B. licheniformis* strain B4089 using antiSMASH. Putative biosynthetic genes are presented in green, additional biosynthetic genes in brown, transport, and regulation-related genes in blue, and resistance genes in red

**Fig. 6:**
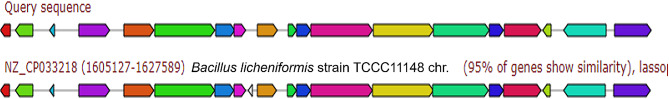
Biosynthetic gene cluster arrangement of Lasso peptide of *Bacillus haynesii* isolate MZ922052 (query sequence) compared to the homologous (95% identity) biosynthetic gene cluster of *B. licheniformis* strain TCCC 11,148 using antiSMASH. Putative biosynthetic genes are presented in green, additional biosynthetic genes in brown, transport, and regulation-related genes in blue, and resistance genes in red

**Fig. 7:**
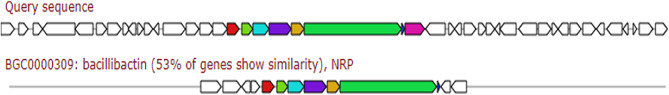
Biosynthetic gene cluster arrangement of bacillibactin of *Bacillus haynesii* isolate MZ922052 (query sequence) compared to the homologous (53% identity) biosynthetic gene cluster of *B. licheniformis* strain TCCC 11,148 using antiSMASH. Putative biosynthetic genes are presented in green, additional biosynthetic genes in brown, transport, and regulation-related genes in blue, and resistance genes in red

**Fig. 8:**
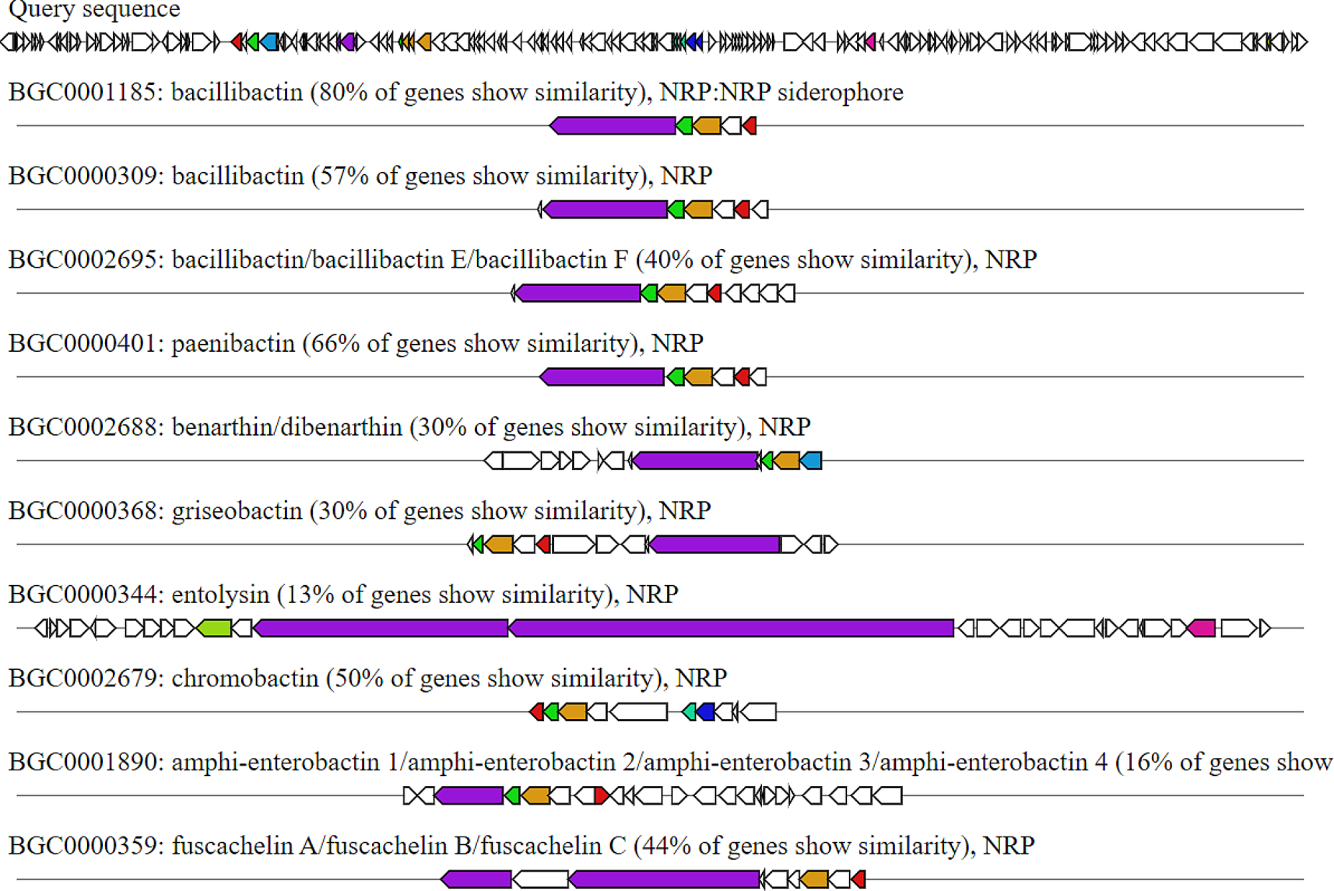
Biosynthetic gene cluster arrangement of bacillibactin of *Bacillus haynesii* isolate MZ922052 (query sequence) compared to the homologous (80% identity) biosynthetic gene cluster using DeepBGC. Putative biosynthetic genes are presented in purple, additional biosynthetic genes in brown, transport, and regulation-related genes in blue, and resistance genes in red

**Fig. 9:**
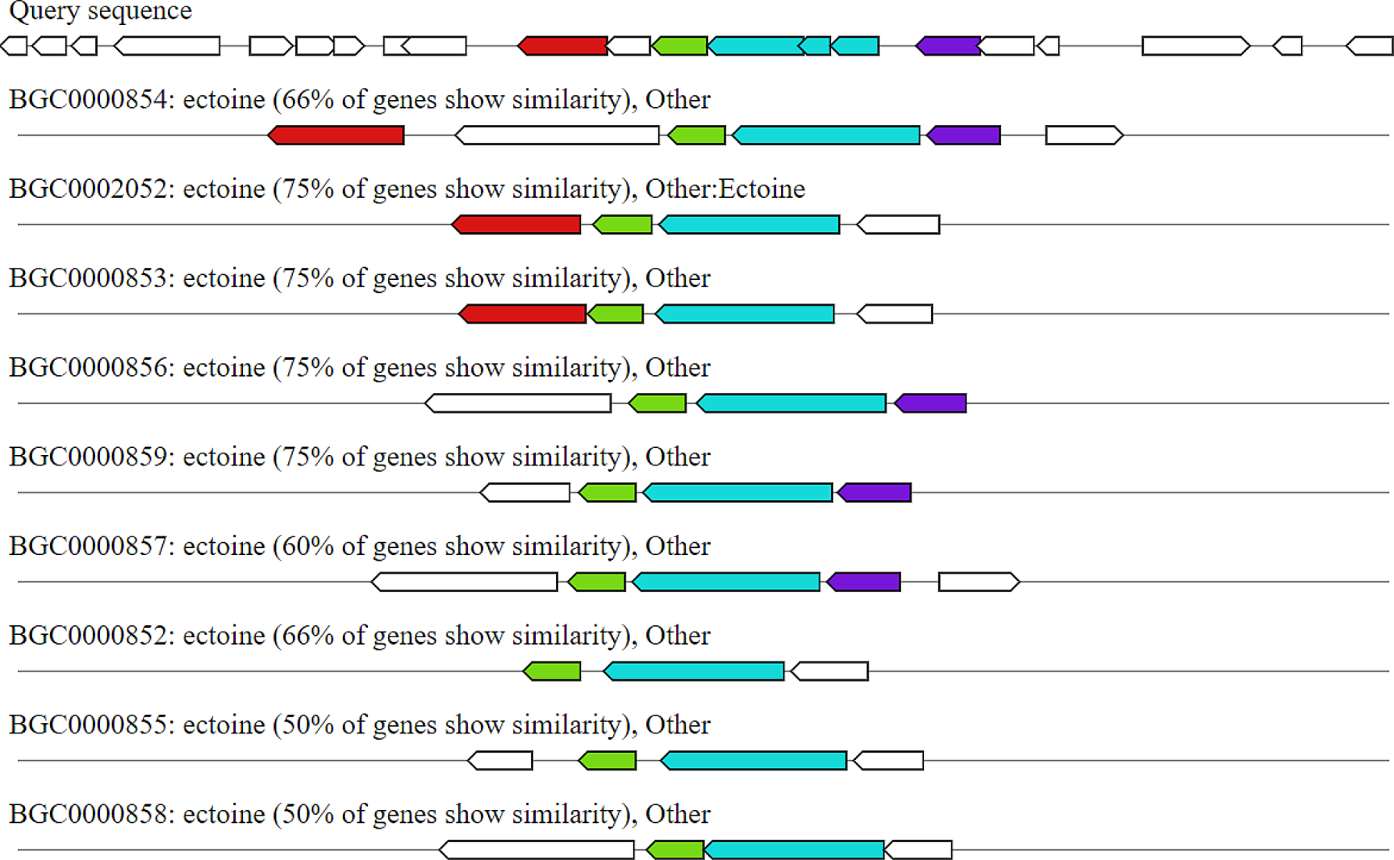
Biosynthetic gene cluster arrangement of ectoine of *Bacillus haynesii* isolate MZ922052 (query sequence) compared to the homologous (66% identity) biosynthetic gene using DeepBGC. Putative biosynthetic genes are presented in blue, additional biosynthetic genes in purule, transport, and regulation-related genes in green, and resistance genes in red

Secondary metabolite gene analysis of *B. haynesii* isolate MZ922052 revealed the presence of the biosynthetic gene clusters of seven secondary metabolites as follows:

- **Lantibiotics**: belong to a class of polycyclic peptide antibiotics. They are characterized by the presence of methyllanthionine or thioether amino acid lanthionine, as well as 2-aminoisobutyric acid and the unsaturated amino acid dehydroalanine. They are ribosomal synthesized and post-translationally modified peptides. (Gene cluster 100% similar to gene cluster producing the siderophore lichenicidin VK21 A1/A2) (Fig. 1).

b) **Traditional (multi-)modular non-ribosomal peptide synthases**: these peptides are structurally and functionally different peptides that have important medical applications. (Gene cluster 100% similar to gene cluster producing lichenysin) (Fig. 2).

c) **Beta-lactone-containing protease inhibitors**: They are efficient biochemical probes and possible leads for new antimicrobial agents. (Gene cluster 53% similar to gene cluster producing the fengycin) (Fig. 3).

d) **Terpene**: Terpenes are a major biosynthetic factory for steroids. They are natural products of essential oils of plentiful flowers and plants. Terpenes have the formula (C_5_H_8_) ^n^ (Fig. 4).

e) **Bacteriocin**: Toxin peptide or protein in nature. They inhibit the growth of bacteria that are closely related or similar. They are diverse concerning structure, function, and ecology. Gene cluster showing 100% similarity to *B. licheniformis* strain B4089 (Fig. 5).

f) **Lasso peptide**: The origin of this metabolite is a peptide in nature that is the origin of many compounds used in medicine as receptor blocking action, inhibition of enzymes, and antimicrobial (Fig. 6).

g) **Traditional (multi-)modular non-ribosomal peptide synthases**. These are biocatalysts to compile diverse peptides of valuable medicinal relevance. This catalysis occurs by utilization of complex stereospecific and regiospecific reactions (Gene cluster 53% similar to gene cluster producing bacillibactin) (Fig. 7).

h) **Bacillibactin**. This is a catechol-based siderophore with considerable broad-spectrum antimicrobial activity. The gene cluster of *B. haynesii* isolate MZ922052 was analyzed using deepBGC and showed about 80% similarities to the bacillibactin biosynthetic gene cluster produced by several members of Bacillus species as displayed in Fig. 8.

i) **Ectoine.** It is a protective substance produced by several bacterial species to allow them to withstand extreme osmotic conditions. The biosynthetic gene cluster of *B. haynesii* isolate MZ922052 was analyzed using deepBGC and it showed 66% similarity to other ectoine biosynthetic gene clusters produced by various microbial species as shown in Fig. 9.

### Cheminformatics

The cheminformatics analysis of the known secondary metabolites produced from the *B. haynesii* isolate MZ922052 has been carried out and the lacticin 3147 structural peptides Ltnα and Ltnβ were used as templates for structure prediction. Results revealed the conservation of the lanthionine-containing peptide antibiotic (lantibiotic) for the lichenicidin (Fig. S4) The 2D structures of lichenysin and fengycin (a derivative of oxapentanoic acid) were carried out and the molecular weight of each molecule was computed using PubChem 2.1 to be 1021.3 and 1463.7, respectively (Figs. S5 and S6).

## Discussion

The continuous evolution of microbes and the accelerated rise in antimicrobial resistance is a pressing need for the discovery of new antimicrobial compounds. Natural resources have always provided solutions to produce several natural products such as antimicrobials(Hutchings et al. 2019). Soil is a habitat for a diverse population of microbes with the potential for new metabolites that have not been discovered yet (Vehapi et al. 2023; Mahjoory et al. 2023). Some of these metabolites are produced as survival mechanisms against microbes present in their environments that we can utilize as antimicrobials. In this study, *B. hayensii* MZ922052 was isolated from soil Luxor Garden, Egypt, and showed antibacterial activity against MDR *K. pneumoniae*, *S. epidermidis*, VRSA and MDR *E. coli*, and a standard and a clinical isolate of *C. albicans.* Peng et al. (2023) isolated a novel bacteriocin from *B. haynesii* which was safe and inhibited the growth of *Propionibacterium acne.* Peng et al. (2023) stated that the bacteriocin was strongly active against Gram-positive bacteria and to our knowledge Peng et al. study is the only study that was done on *B. haynesii* as a producer of antimicrobial metabolite (Peng et al. 2023).

Compared to other studies, *B. licheniformis* collected from Hashemite University Campus area, Jordan, inhibited *Streptococcus pneumoniae* ATCC 6303, *S. aureus* ATCC 11,632, *Proteus mirabilis* ATCC and *Enterobacter cloacae* ATCC 13,182, but showed no activity against *E. coli* ATCC 10,145 and *Salmonella* Typhi ATCC 13,076 (Berić et al. 2014b). A study done on *B. licheniformis* DSM 13 to detect novel antibiotic gene clusters showed antibacterial action in Gram-positive bacteria, like *B. subtilis, S. aureus, Micrococcus luteus, S. simulans, Streptococcus pyogenes*, enterococci, but showed no activity against Gram-negative bacteria (Dischinger et al. 2009). Another example that showed the potential of the *Bacillus*, a study on *B. paralicheniformis* UBBLi30 that was isolated from traditional fermented food in India showed inhibition against *Micrococcus luteus* (and its biofilm formation), *S. aureus, Streptococcus pyogenes, Propionibacterium acnes* and showed no inhibitory effect on *C. albicans, E. coli*, and *P. aeruginosa* (Ahire et al. 2020).

Secondary metabolites in the present research were described by applying TLC and LC/MS and the determination of polarities of metabolites and retention factor (RF) were determined by TLC Eltokhy et al. 2021a, b). Wide diverse compounds were found in the cell-free extract of ethyl acetate (results not shown) as reported by LC/MS. AntiSMASH analysis applied to the sequences provided guided us to the nature of the antibacterial secondary metabolites gene clusters. We could not do the correlation between the inhibitory compounds identified by LC/MS and secondary metabolite gene clusters as all peaks of LC/MS were below 1000 m/z mass, while all secondary metabolites identified by antiSMASH had mass above 1000 m/z.

AntiSMASH analysis showed the presence of seven different antimicrobials including licheniciden (polypeptide lanthibiotic), lichenysin, fengycin, bacteriocin, Lasso peptide, and bacillibactin. The lichenicidin showed 100% similarity to lichenicidin VK21 A1/lichenicidin VK21 A2. The lantibiotic is a peptide antibiotic (lanthionine) that is active on Gram-positive bacteria. Depolarization of the bacterial-energized cytoplasmic membrane is the basis for its bactericidal activity, and this is initiated by the formation of aqueous transmembrane pores (Panina et al. 2023). Our results show that ethyl acetate extract of fermentation products of *Bacillus* sp. have strong growth inhibition against Gram-positive *S. aureus* and VRSA. In silico studies and whole genome sequencing in addition to microbiological studies have proved the presence of gene clusters for lichenicidin production by our isolated *Bacillus* strain.

Furthermore, the obtained metagenome sequence was analyzed using DeepBGC software to detect the presence of secondary metabolite biosynthetic gene clusters as previously described (Hannigan et al. 2019) Our results showed the presence of the biosynthetic gene cluster of two important active metabolites namely, bacillibactin (broad-spectrum antibacterial activity) (May et al. 2001) and ectoine (osmolyte substance) (Peters et al. 1990). The action of action is favorable for bacterial growth under extremely unfavorable condition like high salt concentration and accordingly, it can act as a new target for the development of antibacterial agents like the natural antioxidant staphyloxanthin produced by *Staphylococcus aureus* (Elmesseri et al. 2022). It was previously reported that the bacillibactin class of antibiotics was isolated from marine *Bacillus* species and biochemically identified to have a promising broad-spectrum antibacterial activity (Chakraborty et al. 2022). To the best of our knowledge, this is the first report about identifying the bacillibactin biosynthetic gene cluster in *B. haynesii.*

A large multi-modular biocatalyst called lichenysin. This biocatalyst synthesizes structurally and functionally varied peptides with significant medical applications using intricate stereospecific and regiospecific reactions. It is a potent biosurfactant lipopeptide that prevents the formation of bacterial biofilm. Lichenysin is much more potent than surfactin which is produced by *B. subtilis* (Gudiña and Teixeira 2022). Also, lichenycin has low toxicity which could be used safely. The gene cluster for lichenycin shows 100% similarity to the gene cluster producing lichenysin which predicts our strain to be a source of lichenysin (Coronel-León et al. 2016). A third secondary metabolite which is predicted to be beta-lactone contains protease inhibitors: these are a great biochemical probe and possible source of antibacterial drugs. The predicted compound has 53% similarity to fengycin which is a fungicide used in agriculture (Sur et al. 2018). Fengycin is a cyclic lipopeptide that acts effectively against bacteria and fungi (Sur et al. 2018). The activity of the fermentation ethyl acetate extract or dichloromethane showed very little against *Candida*. This could be due to fengycin not being extracted by the two solvents used. Extraction of fengycin needs to be precipitated first by hydrochloric acid and then extracted with methanol (Lin et al. 2020).

The fourth secondary metabolite predicted by metagenomic analysis is bacteriocin. These are peptides or proteinaceous toxins secreted by bacteria to prevent the growth of related or similar bacterial strain(s). These compounds vary in function, structure, and ecology. They resemble paramecium and yeast microbicidal factors (Benítez-Chao et al. 2021). The metagenomic analysis shows that it is 100% similar to that of *B. licheniformis* strain B4089. Guo et al. (2012) isolated a new strain of *B. licheniformis* from soil that produced a bacteriocin-like substance that was characterized by broad-spectrum antibacterial activity (Guo et al. 2012). The fifth secondary metabolite predicted by metagenomics is the Lasso peptide. Natural products of peptide origin are synthesized by the ribosomes and modified after translation (RiPPs) and identified by having a thread-like structure (Cao et al. 2021). They often are prolific origin of substances with medical relevance. These substances exhibit a variety of intriguing biological actions, including antimicrobial, inhibition of enzyme activity, and blocking receptor activities (Hegemann et al. 2015). Our strain showed 95% similarity to the *B. licheniformis* strain TCCC 11,148 gene cluster for biosynthesis of Lasso peptide. This aligning result indicates that the present strain acquired different posttranslational modification than that of *B. licheniformis* strain TCCC 11,148.

The sixth metabolite is a multi-modular non-ribosomal peptide synthase which is a big multi-modular biocatalyst that uses complex stereo and regiospecific reactions to construct functionally and structurally several peptides that possess significant medical uses. Chakraborty et al. isolated four homologous siderophore types of bacillibactin from marine bacteria *B. amyloliquefaciens* MTCC 12,713 (Chakraborty et al. 2022). *P. aeruginosa, K. pneumoniae*, vancomycin-resistant *Enterococcus faecalis*, and MRSA were among the drug-resistant bacteria against which it showed possible inhibitory effects. Gene clusters of the previous bacteria were characterized by sequencing of the entire genome of *B*. *amyloliquefaciens* MTCC 12,713. In our study bacillibactin showed 53% similarity to gene cluster producing bacillibactin. This might be a novel derivative of bacillibactin which needs further isolation and characterization. The last gene cluster represents terpenes, with general formula (C5H8)n, these are also compounds of natural origin. Terpenes are also major biosynthetic building blocks for steroids. Terpenes and their derivatives have antibacterial activity against sensitive and MDR bacteria by cell rupture and inhibiting the synthesis of both proteins and DNA (Guimarães et al. 2019). Accordingly, the future perspective of this research is to produce and optimize the production of the respective seven active metabolites and test their activities in more detail. One of the promising approaches to optimize the production of certain microbial metabolites is via the use of various models implemented for statistical optimization. This approach has been successfully used for the production optimization of various secondary antibacterial metabolites such as paromomycin (Ibrahim et al. 2019; El-Housseiny et al. 2021; Ibrahim et al. 2023, antifungal metabolites (El-Sayed et al. 2020), biosurfactants (El-Housseiny et al. 2016, 2020), probiotics against life-threatening pathogens (Mansour et al. 2018), medically-used enzymes such as L-asparaginase (Darnal et al. 2023), enterokinase, (Ebrahimifard et al. 2022), staphylokinase (Shariati et al. 2022), and carbohydrases (Kaur et al. 2021)using the response surface methodology and multifactorial design. Therefore, this approach could be a promising tool for the optimization procedures of the potential antibacterial metabolite produced by *B. haynesii* MZ922052. In conclusion, metagenomic nanopore sequence analysis of soil coupled with conventional screening methods has been carried out and was shown to be very helpful in identifying new antimicrobial metabolites and their respective biosynthetic gene clusters produced by the soil microbiota. *B. haynesii* MZ922052 was recovered in this study via conventional screening method coupled with nanopore metagenome screening and showed promising broad-spectrum antibacterial activities against various clinically relevant pathogens. Metagenomic analysis of the respective soil isolate revealed conservation of the biosynthetic gene clusters of seven valuable antibacterial metabolites such as lichenicidin, lichenysin, fengycin. major terpenes, bacteriocin, Lasso peptide, and bacillibactin. This is the first report for identifying the bacteriocin, lichenysin, and fengycin biosynthetic gene clusters in *B. haynesii* MZ922052. Future studies should be conducted to optimize the production of the respective metabolites and obtain them in pure forms followed by characterization and clinical evaluation for their potential use in humans.

### Electronic supplementary material

Below is the link to the electronic supplementary material.

Supplementary Material 1

## Data Availability

and Material. All data generated or analyzed during this study are included in this published article and supplementary file. The 16 S ribosomal RNA is available at NCBI GenBank database under the accession code, MZ922052 https://www.ncbi.nlm.nih.gov/nuccore/MZ922052.1/ (accessed on 16 December 2024). The metagenomics sequences were deposited in the NCBI GenBank sequence Archives under accession number PRJNA1064698 (https://www.ncbi.nlm.nih.gov/sra/PRJNA1064698 (accessed on 16 December 2024).

## Declarations

**Ethical approval**.
