# Supplementary material for: Metagenomic nanopore sequencing for exploring the nature of antimicrobial metabolites of Bacillus haynesii
Source: AMB Express. 2024 May 4;14:52. doi: 10.1186/s13568-024-01701-8 (PMC11069495; doi:10.1186/s13568-024-01701-8)
Supplement: Supplementary file 1 — Supplementary Material 1 [file 13568_2024_1701_MOESM1_ESM.docx]

**Metagenomic Nanopore Sequencing for exploring the nature of antimicrobial metabolites of *Bacillus haynesii***

Mohamed A. Eltokhy ^1^, Bishoy T. Saad ^2^, Wafaa N. Eltayeb ^1^, Mohammad Y. Alshahrani ^3^, Sahar M.R. Radwan^4^, Khaled M. Aboshanab ^4,^* and Mohamed S. E. Ashour ^7^

^1^Department of Microbiology, Faculty of Pharmacy, Misr International University (MIU), Cairo 19648, Egypt; [mohammad.ashraf@miuegypt.edu.eg](mailto:mohammad.ashraf@miuegypt.edu.eg); [wafaa.eltayeb@miuegypt.edu.eg](mailto:wafaa.eltayeb@miuegypt.edu.eg)

^2^Department of Bioinformatics, HITS Solutions Co., Cairo 11765, Egypt; [bishoyth@hitssolutions.com](mailto:bishoyth@hitssolutions.com)

**^3^**Department of Clinical Laboratory Sciences, College of Applied Medical Sciences, King Khalid University, P.O. Box 61413, Abha, 9088, Saudi Arabia. [moyahya@kku.edu.sa](mailto:moyahya@kku.edu.sa)

**^4^**Department of Microbiology and Immunology, Faculty of Pharmacy, Al-Azhar University (Girls), Cairo 11651, Egypt. [saharradwan561.el@azhar.edu.eg](mailto:saharradwan561.el@azhar.edu.eg)

^5^Department of Microbiology and Immunology, Faculty of Pharmacy, Ain Shams University, Organization of African Unity St., Cairo 11566, Egypt. [aboshanab2012@pharma.asu.edu.eg](mailto:aboshanab2012@pharma.asu.edu.eg)

**^7^**Department of Microbiology and Immunology, Faculty of Pharmacy, Al-Azhar University (Boys), Cairo 11651, Egypt; [seifashour@hotmail.com](mailto:seifashour@hotmail.com)

***Corresponding Author**: **Khaled M. Aboshanab (PhD)**

Address: Department of Microbiology and Immunology, Faculty of Pharmacy, Ain Shams University, organization of African unity St. PO: 11566, Abbassia, Cairo, Egypt.

E-mail: [aboshanab2012@pharma.asu.edu.eg](mailto:aboshanab2012@pharma.asu.edu.eg)

Tel: (202)28429040

Mobile: (002)01007582620

Fax: (202)24051107

<https://orcid.org/0000-0002-7608-850X>

**
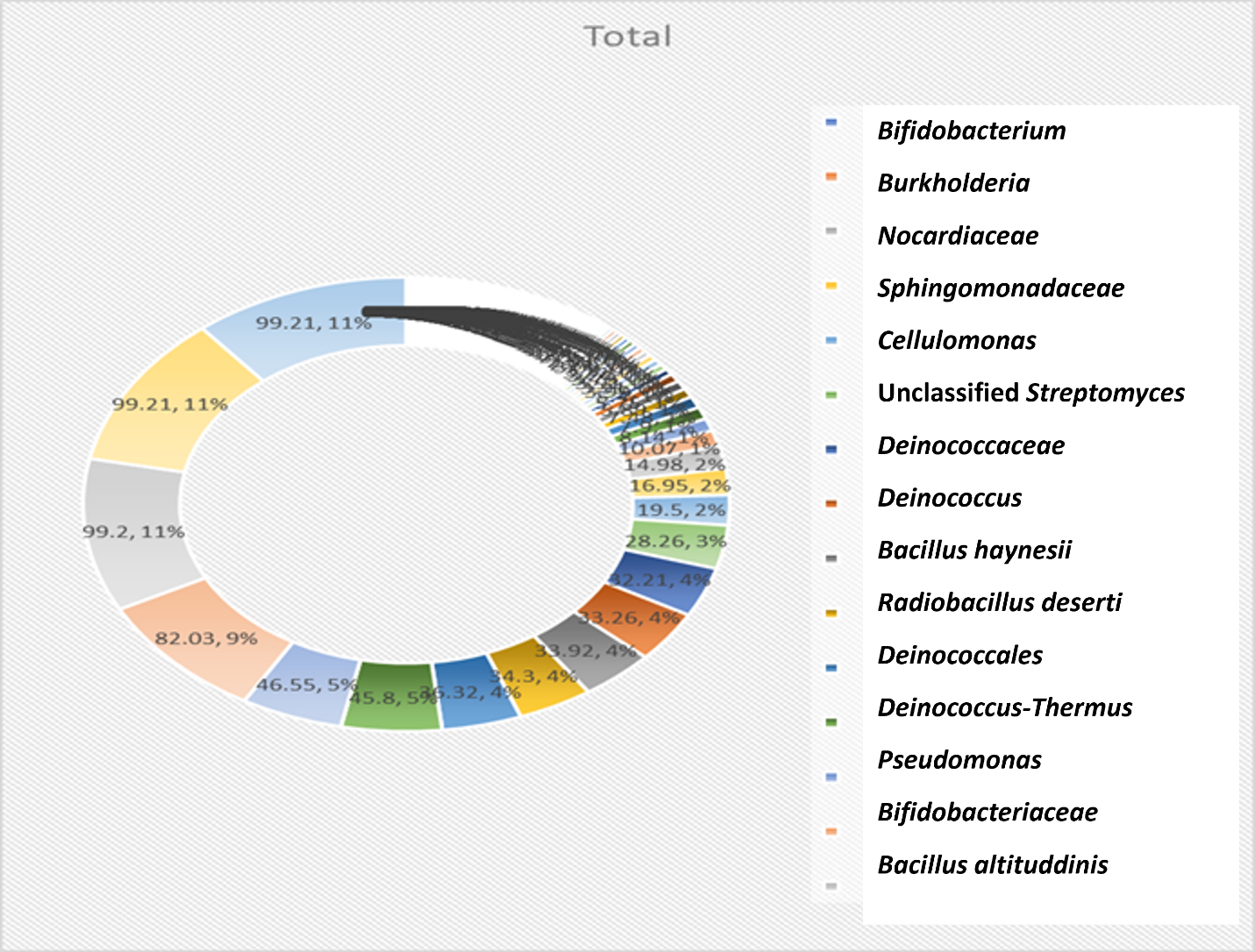
**

**Fig. S1**. The percentage of fragments covered by the clade rooted at this taxon, percent abundance of the bacterial phylum located in the soil.


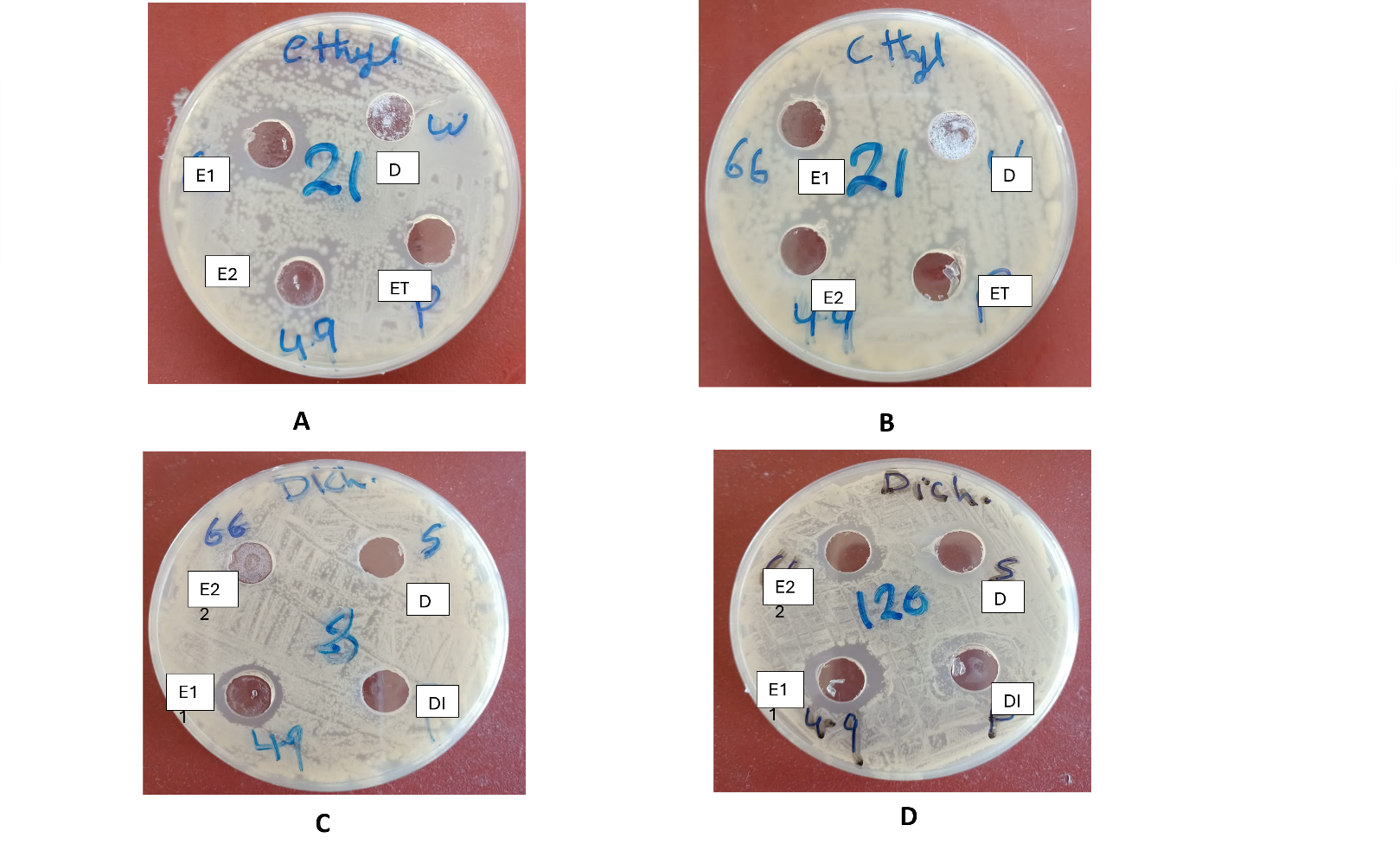


**Figure S2**. Agar diffusion for evaluating the antimicrobial activities of ethyl acetate extract of *B. haynesii* isolate MZ922052 against (A) MDR *K. pneumoniae* clinical isolate (KP1) (B) *C. albicans* clinical isolate (CA1) and dichloromethane extract against (C) vancomycin resistant *Staphylococcus aureus* (VRSA1)and (D) MDR *Escherichia coli* (EC1). D, DMSO solvent as a control, ET, ethyl acetate solvent as a control, DI, dichloromethane as a control, E1 (100% of the extract), E2 (50% diluted extract with DMSO).


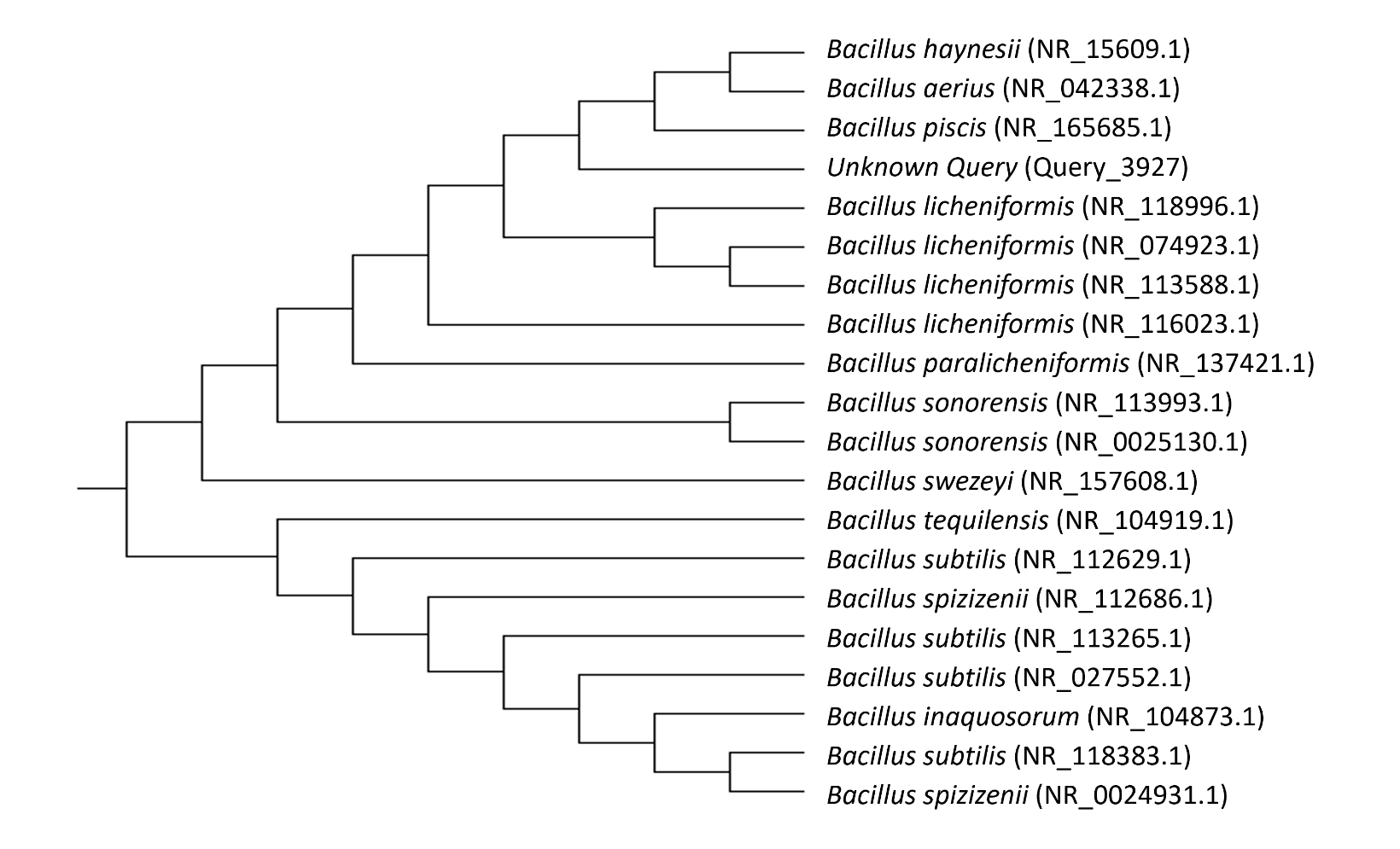


**Figure S3**. Molecular phylogenetic analysis of the 16S ribosomal RNA of the *Bacillus* isolate SS10 (Query) with homologous 16S ribosomal RNA sequences of various *Bacillus* sp. Log-Expectation through Multiple Sequence Comparisons using MUSCLE (MUSCLE, <https://www.ebi.ac.uk/Tools/msa/muscle>) (accessed on 18 December 2023). Bootstrap analysis (1000 replicates) was applied for inferring phylogenetic trees.


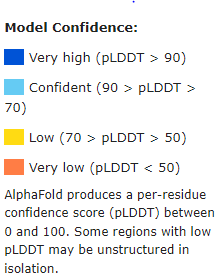


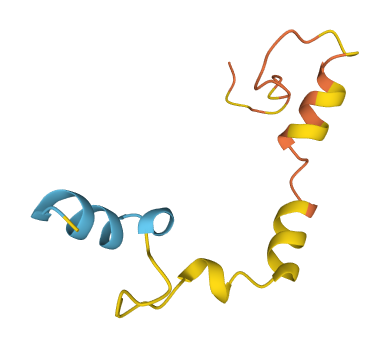


**Fig. S4** 3D structure of ichenicidin: lanthionine-containing peptide antibiotic (lantibiotic)predicted with AlphaFold. lacticin 3147 structural peptides Ltnα and Ltnβ were used as templates for Structures prediction. doi: [10.1128/AEM.00730-09](https://dx.doi.org/10.1128%2FAEM.00730-09)


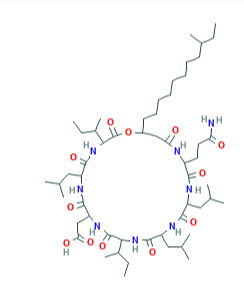


**Fig. S5**. 2D structure of lichenysin. Molecular weight is computed by PubChem 2.1 to be 1021.3

**
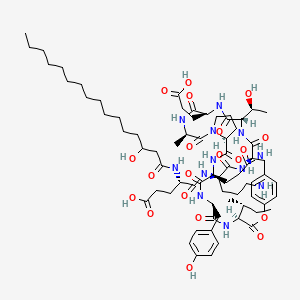
**

**Fig. S6**. 2D structure of fengycin. A derivative of oxapentanoic acid showing molecular weight 1463.7 computed by PubChem 2.1
